# Predicting and containing epidemic risk using on-line friendship networks

**DOI:** 10.1371/journal.pone.0211765

**Published:** 2019-05-16

**Authors:** Lorenzo Coviello, Massimo Franceschetti, Manuel García-Herranz, Iyad Rahwan

**Affiliations:** 1 Google Inc., Pittsburgh, PA, United States of America; 2 Department of Electrical and Computer Engineering, University of California San Diego, La Jolla, CA, United States of America; 3 Unicef, New York, NY, United States of America; 4 Media Laboratory, Massachusetts Institute of Technology, Cambridge, MA, United States of America; University of Bristol, UNITED KINGDOM

## Abstract

To what extent can online social networks predict who is at risk of an infection? Many infections are transmitted through physical encounter between humans, but collecting detailed information about it can be expensive, might invade privacy, or might not even be possible. In this paper, we ask whether online social networks help predict and contain epidemic risk. Using a dataset from a popular online review service which includes over 100 thousand users and spans 4 years of activity, we build a time-varying network that is a proxy of physical encounter between its users (the encounter network) and a static network based on their reported online friendship (the friendship With computer simulations, we compare stochastic infection processes on the two networks, considering infections on the encounter network as the benchmark. First, we show that the friendship network is useful to identify the individuals at risk of infection, despite providing lower accuracy than the ideal case in which the encounters are known. This limited prediction accuracy is not only due to the static nature of the friendship network because a static version of the encounter network provides more accurate prediction of risk than the friendship network. Then, we show that periodical monitoring of the infection spreading on the encounter network allows to correct the infection predicted by a process spreading on the friendly staff ndship network, and achieves high prediction accuracy. Finally, we show that the friendship network contains valuable information to effectively contain epidemic outbreaks even when a limited budget is available for immunization. In particular, a strategy that immunizes random friends of random individuals achieves the same performance as knowing individuals’ encounters at a small additional cost, even if the infection spreads on the encounter network.

## Introduction

The forecast and containment of epidemics is a central theme in public health [1–4]. Events such as the recent ebola epidemic constantly drive the attention and resources of institutions such as the World Health Organization, governments, and researchers [5–7]. Beside biological epidemics, the study of infectious processes is of broad interest as it models the spread of information, behaviors, cultural norms, innovation, as well as the diffusion of computer viruses [8–11]. As it is impossible to study the spread of infectious diseases through controlled experiments, and thanks to advancements in computation, modeling efforts have prevailed [12–14].

The spread of an infection over a real-world network is determined by the interplay of two processes: the structural dynamics *of* the network, whose edges change over time, and the infection dynamics *on* the network, whose paths are constrained by the realization of the former process. When the two dynamics operate at comparable time scales, the time-varying nature of the network cannot be ignored [15–19], specifically devised control strategies are necessary [20], and aggregating the dynamics of the edges into a static version of the network can introduce bias [18, 21]. Empirical work suggests that bursty activity patterns slow down spreading [22–24], but temporal correlations seem to accelerate the early phase of an epidemic [25, 26].

Knowledge of the patterns of human encounter is fundamental for monitoring and containing outbreaks. Various sources of data can be used as a proxy of physical encounter, for example check-ins on social networking platforms, traffic records, phone call records, and wearable sensor data constitute examples [27–29]. However, pervasive and detailed information is rarely available and might be expensive and unpractical to collect (as in the case of sensor technologies), prone to errors (as in the case of survey data), and collection might invade privacy [30–32].

There has been a growing interest in the role of indirect transmission in networks and control measures for reducing epidemic sizes [33–36]. In the absence of information about human encounter, social network information obtained via mining of massive on-line social platforms might be useful to design strategies for containment of epidemic outbreaks. To what extent can online social networks predict who is at risk of infection? How can information about such relationships help monitor and contain epidemic outbreaks? Recent work has suggested that communication traces obtained from mobile phones might help reduce the expected size of an epidemic [37]. In addition, networks generated from wearable sensor measurements, diaries of daily contacts, online links and self-reported friendship present similar structural properties [38]. However, it is unclear whether these global structural properties are representative of similarities in epidemic processes at the microscopic scale. In general, it is not even clear whether friendship can be considered a reliable proxy of physical encounter, as a process spreading from an initial seed, or “patient zero”, can only reach the nodes within its *set of influence* through paths that respect time ordering [39].

In this work, we study the prediction of epidemic risk at the individual level, using computer simulation. In particular, we use the friendship ties between the individuals in a social network to predict who has a high probability of becoming infected, given an infection driven by physical encounter initiated at a known infection seed. The ability of identifying who is at risk of infection is critical to inform containment procedures, such as the immunization of particular groups, or the mobilization of treatment facilities to specific communities at risk.

Using a dataset from the popular online review service Yelp (we consider the Yelp Dataset Challenge dataset, Round 5: www.yelp.com/dataset_challenge) which includes over 100k users and spans 4 years of activity, we build a time-varying network that is a proxy of physical encounter between users and a static network based on their reported friendship—the encounter network and the friendship network. For comparison, we also consider a static version of the encounter network, in which temporal information is ignored. Through computer simulation, we study Susceptible-Infected processes [40] spreading on the different networks and compare the sets of infected individuals, assuming that real infections spread on the encounter network and that the friendship network is available and used for prediction purposes. Considering simulated infections on the time-varying encounter network as the benchmark, we quantify how the friendship network and the static version of the encounter network provide prediction of individual-level risk.

Given epidemic processes spreading independently on the encounter and friendship networks but initiated at the same seed, we show that the friendship network contains useful information for predicting epidemic risk at the individual level. In particular, the set of nodes infected by processes spreading on the friendship network approximates those infected by processes spreading on the encounter network substantially better than random guessing. In addition, given epidemics spreading on the encounter network, a node’s probability of becoming infected decreases with its distance from the infection seed on the friendship network. These are important results, as in practice it might be feasible to track friendship or other forms of static relationship, while infeasible to track or predict physical encounter.

However, the prediction accuracy obtained with the friendship network does not come close to an ideal case in which the encounters are known (which is usually not the case in practice). Even if the stochasticity of the infection process certainly contributes to the unpredictability of risk at the individual level, the difference between the two networks plays a major role. In fact, two independent infections spreading on the encounter network and started at the same seed have on average substantially higher similarity than two infections spreading on the two different networks. This result is driven not only by the static nature of the friendship network as opposed to the time-varying nature of the encounter network, because a static version of the encounter network provides more accurate prediction of risk than the friendship network.

From a practical point of view, reported friendship ties can help monitor and contain epidemic outbreaks. On the one hand, we show that periodical, but even relatively infrequent, observations of the benchmark infection boosts the accuracy of risk prediction using the friendship network. In particular, we consider a scenario in which the encounter network is still unknown, but the set of infected nodes is observed periodically. In the case of real epidemics, reports of the infected population are usually available at regular intervals, daily, weekly or monthly, in the form of situational reports or through case management systems. After each observation, the set of infected individuals estimated by running the process on the friendship network is updated to match the set of individuals infected by a process spreading on the encounter network. By comparing the predicted infected set (obtained with the friendship network and periodical updates) and the benchmark infected set (obtained with the encounter network) immediately before each update, we show that a high level of accuracy is reached and maintained even with infrequent observations.

On the other hand, we show that online friendship ties allow to effectively allocate a limited immunization budget in order to reduce the risk of an outbreak, even if the infection spreads on the encounter network. In particular, we consider the strategy of providing immunization to random friends of randomly selected individuals, motivated by the “friendship paradox” [41–43], according to which the average individual in a network is less connected than their average friend. Compared to a basic strategy that provides immunization to randomly selected individuals, the proposed strategy increases the probability that an infection dies out in its early stages, and always reduces the size of the infected population. Its implementation only requires individuals to name a friend and avoids computing metrics such as degree and centrality. Despite its simplicity, it only requires a relatively small additional cost to provide the same effectiveness as a strategy that immunizes encounters of random individuals (which would therefore require knowledge of the encounter network).

## Methods

### Dataset

The Yelp Dataset Challenge dataset Round 5 (www.yelp.com/dataset_challenge, code at https://zenodo.org/record/2598838#.XJGuuxNKjOQ). consists in 1, 569, 264 reviews and 495, 107 tips to 61, 184 businesses (in 10 cities around the world) posted by 366, 715 users over a period spanning over 10 years. Within this period, we consider 1, 469 consecutive days ranging from 1/1/2011 to 1/8/2015, as reviews before 2011 are less numerous. Each review and tip includes the user who posted it, the reviewed business, and the date it was posted. Yelp users can form friendship ties between each other, and the list of friends of each user is included in the dataset. Time information about the formation of friendship ties is not available. Using the dataset, we define two networks, called the friendship network and the encounter network respectively.

Let *U* be the set of users, *F* ⊆ *U* × *U* be the set of friendship ties, *B* the set of businesses, *T* be the set of days, *R* ⊆ *U* × *B* × *T* be the set of reviews and tips (which we will refer to as reviews). For each user *u* ∈ *U* let *F*_*u*_ ⊆ *U* be the set of friends of *u*. Therefore *F* = ∪_*u* ∈ *U*_{(*u*, *v*) : *v* ∈ *F*_*u*_}. Each review (or tip) *r* ∈ *R* is a triple (*u*, *b*, *t*) where *u* ∈ *U*, *b* ∈ *B*, *t* ∈ *T*.

### The friendship network

**Fig 1:**
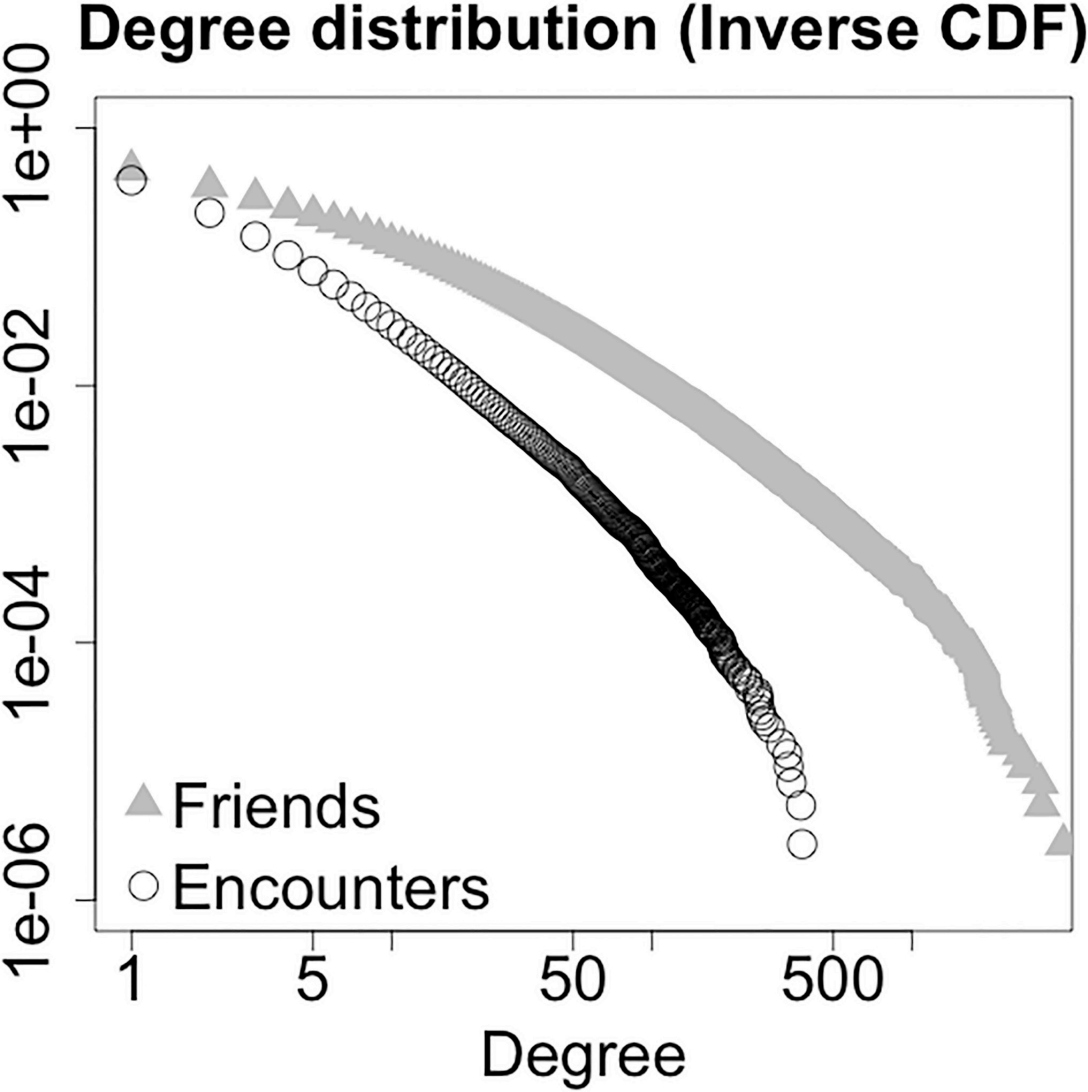
Inverse cumulative distribution function of friend degree (grey triangles) and encounter degree (white circles). The friend degree of a user is defined as their number of friendship ties. The encounter degree of a user is defined as their number of encounters during all period of observation.

Of all users, 174, 100 have at least one friend, with an average number of friends per user, or friend degree, 14.8. The friend degree distribution is shown in Fig 1 (triangles).

Let *N*_*F*_ = (*U*, *F*) be the static friendship network. As we consider processes spreading between connected nodes, connectedness is the key property of the networks. Therefore, we restrict our attention to the giant component, as users outside giant components form small components whose dynamics are not relevant. The giant component defined by friendship includes 168, 923 users (whereas the second largest component has 8 users). In what follows, we will identify *N*_*F*_ with its giant component. Observe that this network is static, as its edges do not change over time.

### The encounter network

The most common vehicle for the spread of infectious diseases is physical contact (rather than friendship) between individuals. Strictly speaking, two users in *U* encountered on a given day *t* if they visit the same business on day *t* at the same time. In the present work, given the data available to us, we consider reviews instead of physical encounter: an edge is active between two users in *U* on day *t* if they posted a review to the same business on day *t*. Real physical encounter requires users to *visit* (rather than review) a business at about the same time, but we assume that the time of a review is a *proxy* of the time of the visit to a business. The data at our disposal does not allow us to derive statistics about how likely a user is to have visited a place over a given time interval preceding posting a review and we know of no publicly available statistics or data about this issue. Our assumption is in part justified by the fact that the element that spreads over a network (e.g., a virus or an opinion) does not necessarily require direct physical contact. For example, in the case of airborne transmission, particles can remain suspended in the air for hours after an infected individuals has occupied a room [44]. In the context of our dataset, after an infected user visits a business, the infection might spread to customers who visit the business later in the day. Also, the virus can infect customers which are not included in the dataset, and from them can infect another user who visits the business in a later moment.

For each *t* ∈ *T*, *U*(*t*) = {*u* ∈ *U* : (*u*, *b*, *t*) ∈ *R* for some *b* ∈ *B*} is the set of users who wrote a review on day *t*. We refer to *U*(*t*) as the active users on day *t*.

For each *t* ∈ *T* and *u* ∈ *U*(*t*), *E*_*u*_(*t*) = {*v* ∈ *U*(*t*), *v* ≠ *u* : (*u*, *b*, *t*) ∈ *R* and (*v*, *b*, *t*) ∈ *R* for some *b* ∈ *B*} ⊆ *U* is the set of encounters of user *u* on day *t* (i.e., users who visited at least one of the businesses visited by *u*). *E*(*t*) = ∪_*u* ∈ *U*_{(*u*, *v*) : *v* ∈ *E*_*u*_(*t*)} ⊆ *U* × *U* is the set of encounters on day *t*.

For each *t* ∈ *T*, let *N*_*E*_(*t*) = (*U*, *E*(*t*)) be the network defined by the encounters on day *t*. Observe that the node set in the definition is *U* rather than *U*(*t*). The *encounter network* is the sequence {*N*_*E*_(*t*)}_*t* ∈ *T*_. As connectedness is the key property in a spreading process, we consider the 133, 038 users who had at least one encounter during *T*.

**Fig 2:**
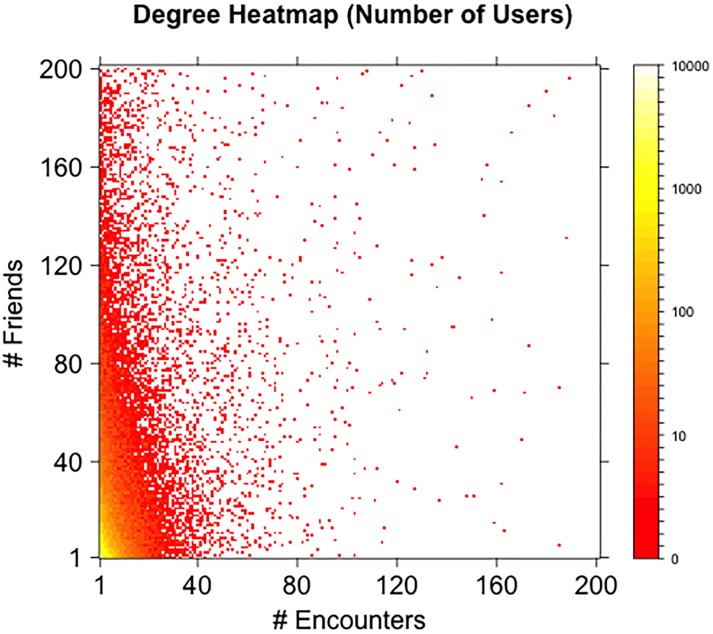
Friends and encounters degree. Heat map of friend degree and encounter degree of all users with at least one friend and one encounter (friend degree and encounter degree are limited to 200 in the plot). The *x*-coordinate and *y*-coordinate on the map represent encounter degree and friend degree respectively. Each (*x*, *y*) coordinate represents the number of users with encounter degree equal to *x* and friend degree equal to *y* (smaller numbers are represented by red color tones, higher numbers by yellow color tones).

**Fig 3:**
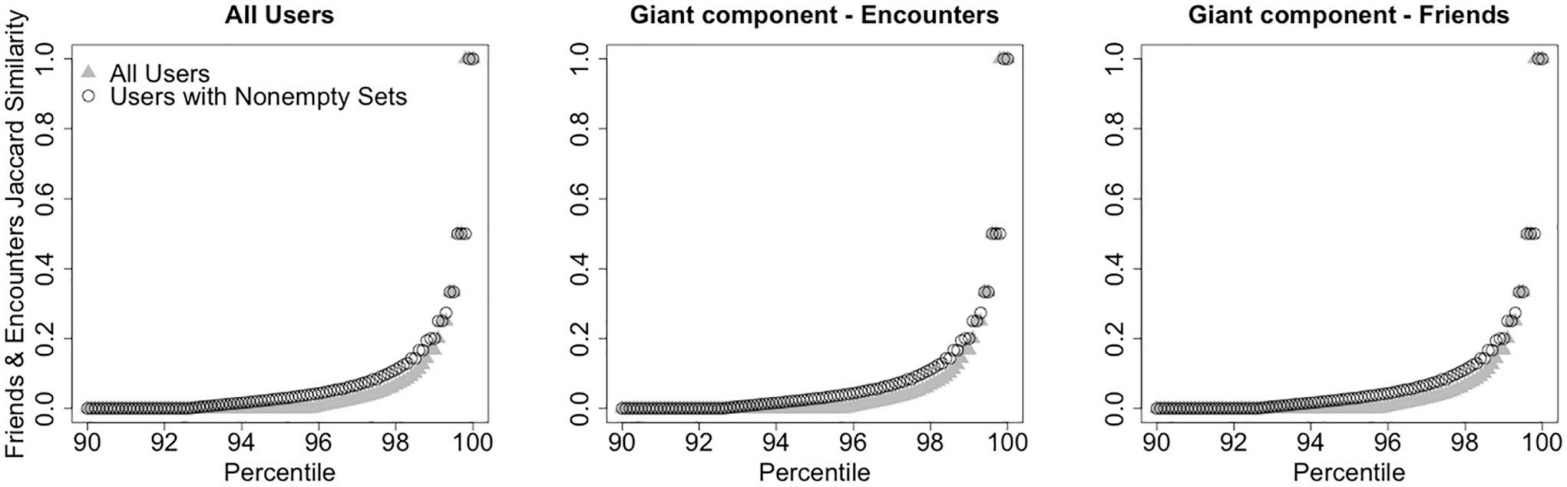
Similarity of friend and encounter sets. Percentile plot of the Jaccard similarity of the set all user’s friends and the set of all user’s encounters. Left: all non-singleton users. Center: users in the giant component of the network defined by all encounters. Right: users in the giant component of the network defined by all friendship ties.

The distribution is shown in Fig 1 (circles). Fig 2 shows a heat map of friend degree and encounter degree of users. The *x*-coordinate and *y*-coordinate on the map represent encounter degree and friend degree respectively. Each (*x*, *y*) coordinate represents the number of users with encounter degree equal to *x* and friend degree equal to *y* (smaller numbers are represented by red color tones, higher numbers by yellow color tones). Despite friend degree and encounter degree are correlated (Pearson product-moment correlation 0.3416, p-value <2.2 ⋅ 10^−16^), the similarity of the sets of the friends and encounters of an individual is low. Fig 3 shows the cumulative distribution function of the Jaccard similarity of the set of friends and the set of encounters of all users in the dataset (left panel), of all users in the giant component of the network defined by all friendship ties (center panel), and of all users in the giant component of the network defined by all encounters (right panel). Considering the 72, 786 users with at least one friend and one encounter, the average Jaccard similarity of their encounter and friend sets is 0.01716, with only 9, 527 of them with a value different than zero. Looking at the giant component of the network defined by all friendship ties, the users with nonzero encounters have average Jaccard similarity of their encounter and friend set of 0.1306, with only 9, 022 users with a nonzero value. Looking at the giant component of the network defined by all encounters, the users with nonzero friends have average Jaccard similarity of their encounter and friend set of 0.112, with only 8, 278 users with a nonzero value. In general, the sets of encounters and of friends of a user can significantly different and often have empty intersection. Despite epidemic processes spreading on the friendship and on the encounter network evolve in a qualitatively similar way, the differences in local connectivity determined by the two definitions of edges might result in very different sets of nodes at risk of infection.

### The static encounter network

To argue that our results are not driven by the static nature of the friendship network as opposed to the time-varying nature of the encounter network, we also consider a static version of the encounter network. Let *E*_*u*_ = ∪_*t*∈*T*_ *E*_*u*_(*t*) ⊆ *U* be the set of encounters of *u* during *T*, and *E* = ∪_*t*∈*T*_∪_*u* ∈ *U*_{(*u*, *v*) : *v* ∈ *E*(*t*)} ⊆ *U* × *U* be the set of encounters between users in *U*. The *static encounter network* is *N*_*E*_ = (*U*, *E*). We restrict our attention to the giant component of the static encounter network, which includes 113, 187 users (whereas the second largest component has 23 users).

We could have considered a weighted version of the static encounter network, where the edge between nodes *u* and *v* has weight *w*_*u*,*v*_ = |{*t* : (*u*, *v*) ∈ *E*(*t*)}|, that is, equal to the number of encounters between *u* and *v* over *T*, and where infection rates are not constant over edges but proportional to weights. Our definition of static encounter network was driven by simplicity, and included an edge between nodes *u* and *v* as long as they encountered at least once over *T*. Such a simple model is motivated by the reason we introduced the static encounter network, that is, to show its increased prediction accuracy with respect to the friendship network, in order to argue that the limits of the friendship network are not only driven by its static nature.

### Infection dynamics

To model the spread of an infectious disease, we consider a Susceptible-Infected (SI) process [40], in which nodes never recover after being infected. Here, we give a general definition of the process that applies to both the static and the time-varying networks defined above. Given a set of nodes V, a set of edges E⊆V×V and a set of time indices T, let ${\left\{N\left(t\right)\right\}}_{t\in T}$ be a sequence of networks, where N(t)=(V,E(t)) with E(t)⊆E. For a static network, E(t)=E for all of t∈T.

Let I(t) denote the set of infected nodes at time *t*, of cardinality *I*(*t*). The infection starts at time *t* = 0 from a set I(0) of infected seeds.

Consider any *t* > 0. The infection spreads from the set of already infected nodes I(t-1) as follows. For each non-infected node v∈V\I(t-1), let $d_{v}\left(t\right)=|\left\{u\in I\left(t-1\right):\left(u,v\right)\in E\left(t\right)|\right\}$, that is, the number of neighbors of *v* at time *t* which are infected at time *t* − 1. Let $B\left(t\right)=\{v\in V\backslash I\left(t-1\right):d_{v}\left(t\right)>0\}$, that is, the set of susceptible nodes at time *t*. We assume that each node *v* ∈ *B*(*t*) gets infected with probability min{*βd*_*v*_(*t*), 1}, where *β* ∈ [0, 1] is the rate of infection.

When *β* = 1 the infection process is deterministic and, at time *t*, all non-infected neighbors of the nodes infected by time *t* − 1 become infected. For finite values of *β*, the infection spreads in a stochastic way. We consider different values of *β* for the different networks, due to their different connectivity (*β* = 0.5 on the encounter network, and *β* = 0.01 on the static networks, unless differently stated).

For the time-varying networks defined above (i.e., the encounter network and the time-varying friendship network), T=T. The infection will propagate for |*T*| time steps, resulting in an infected population I(∈T∈). For static networks (i.e., the friendship network), T=[0,∞) and the infection propagates until I(t)=V (i.e., until the entire population is infected).

Our investigation does not include more general models such as SIR processes (Susceptible-Infected-Recovered), where an infected node recovers from infection with rate *γ* and after recovery cannot spread the infection to its neighbors (from a dynamical point of view, the node is removed from the network). In the considered SI process *γ* = 0 and nodes never recover from infection. We made this decision to focus on the structural properties of the two networks (friendship and encounter) rather then the dynamical properties of the infection processes (infection and recovery rate). We believe that our results extend to SIR models with reasonable values of the parameters *β* and *γ*, but we leave the question to future investigations.

### Infection time

Given a realization of the infection process, for each m∈[0,|V|], let

$$\begin{array}{c} t(m)=\min\{t:I(t)\geq m\}. \end{array}$$

The random variable *t*(*m*) denotes the first time in which at least *m* nodes are infected. Given a realization of the SI process on a time-varying network, let *t*(*m*) = ∞ for M>I(|T|). In what follows, the notation *t*_*A*_(*m*) indicates that nodes on a specific network *A* are considered (e.g. *A* can be the friendship or the encounter network, even if the infection spreads on the encounter network).

### Seed selection

In a static network, seeds are chosen at random and without replacement. In a time-varying network, the infection can start propagating at the first time *t* in which there is an edge between an infected seed and a non-infected node, that is, at time

$$\begin{array}{c} t_{0}\left(I\left(0\right)\right)=\min\left\{t:\exists \left(u,v\right)\in E\left(t\right)\;\text{for}\;\text{some}\;u\in I\left(0\right),v\in V\backslash I\left(0\right)\right\}. \end{array}$$

As a remark, for *β* < 1, it is possible that no node is infected at time *t*_0_. Seeds are selected uniformly at random and without replacement among all nodes v∈V such that *t*_0_({*v*}) ≤ 500, that is, nodes that have a neighbor in the time-varying network by time *t* = 500.

### Real infection and predicted infection

Assuming simulated infections on the time-varying encounter network as the benchmark, we quantify the extent to which the friendship network can predict risk at the individual level. Simulated infections on the static version of the encounter network will serve instead as a comparison, in order to characterize how the loss of temporal information affects prediction accuracy. In other words, we consider infection dynamics on the encounter network as the real infections, and try to predict them by running infection dynamics on the friendship network and on the static version of the encounter network.

## Results

### Epidemic risk and network distance

In this section we show that distance on the friendship network is correlated to epidemic risk. Given and infection initiated at a single seed and spreading on the encounter network, nodes at a shorter distance from the seed on the encounter network have a higher probability of becoming infected. In the rest of the section, we always consider infections spreading on the encounter network and distance defined on the friendship network.

Given nodes *s* and *s*′ in the friendship network, let *d*(*s*, *s*′) denote their distance (i.e., the length of the shortest path connecting them). Given node *s* and an integer *d* > 0, let

$$\begin{array}{c} N_{d}\left(s\right)=\left\{s:d\left(s,s^{\prime }\right)=d\right\} \end{array}$$

be the set of nodes at distance *d* from *s*, and let *n*_*d*_(*s*) be its cardinality. *N*_1_(*s*) and *n*_1_(*s*) denote the set of neighbors and the degree of *s*, respectively.

Let *i* denote an infection process, and *s*_*i*_ the selected seed. Given an infection initiated at a seed *s*_*i*_ until time *T*, let $I(s_{i})$ be the set of infected nodes at time *T*. For each *d* > 0 let

$$\begin{array}{c} I_{d}\left(s_{i}\right)=I\left(s_{i}\right)\cap N_{d}\left(s\right) \end{array}$$

be the set of infected nodes that are at distance *d* from *s*_*i*_ on the encounter network. The infection fraction of nodes at distance *d* from *s*_*i*_ is defined as

$$\begin{array}{c} r_{d}\left(s_{i}\right)=\frac{|I_{d}\left(s_{i}\right)|}{n_{d}\left(s\right)}. \end{array}$$

The empirical average of *r*_*d*_(*s*_*i*_) over *S* simulations is given by

$$\begin{array}{c} {\bar{r}}_{d}=\frac{1}{S}\sum_{i=1}^{S}r_{d}\left(s_{i}\right), \end{array}$$

and represents the risk of becoming infected if the seed is at distance *d*.

**Fig 4:**
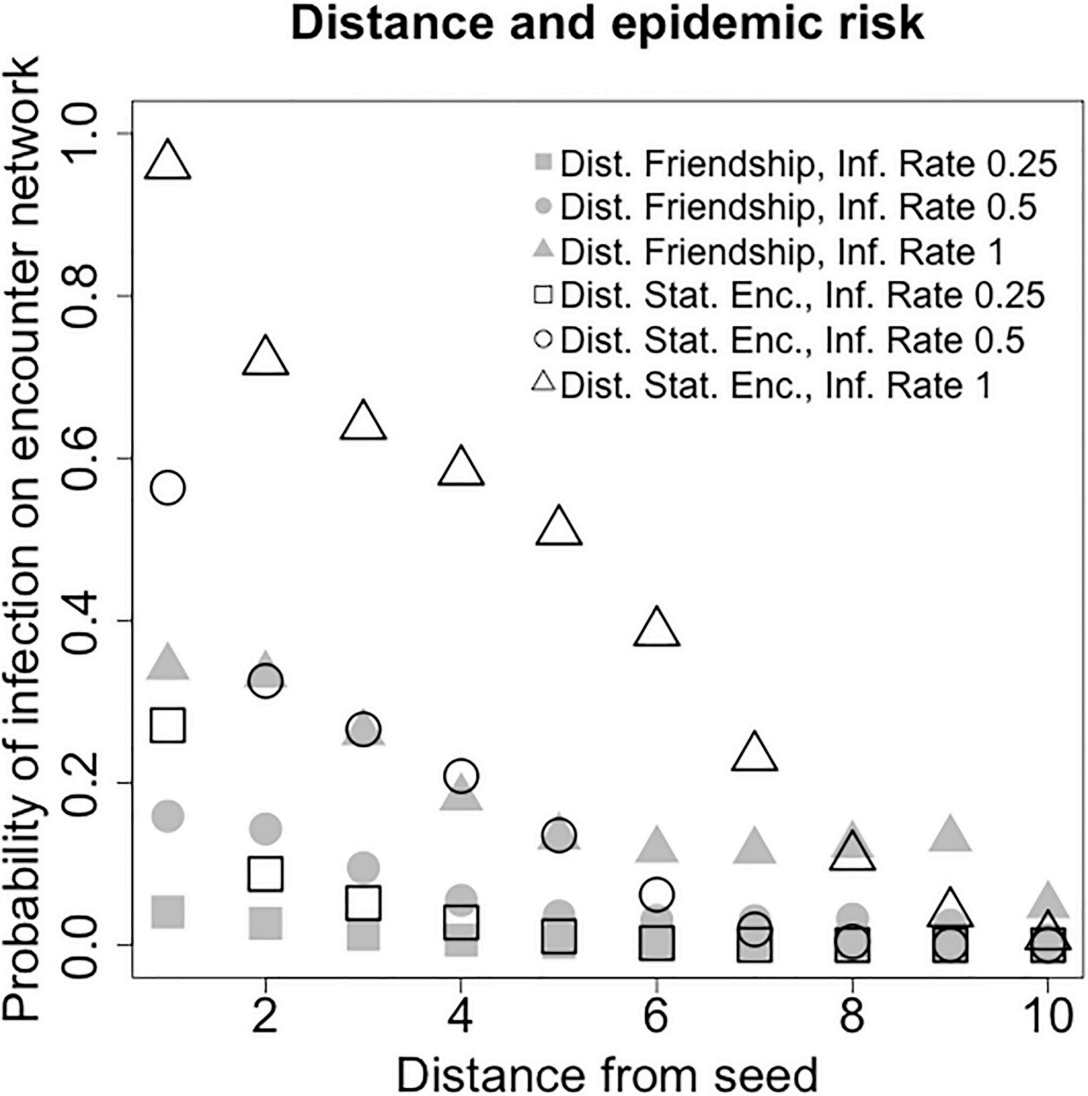
Risk of infection on the encounter network versus distance from the infection seed. Both distance from the seed on the friendship network and distance from the seed on the static encounter network are considered. For each value of the infection rate *β* and each notion of distance from the seed, 10,000 simulations on the encounter network initiated at random single seeds are run. The *x*-axis plots the distance *d* from the seed, the *y*-axis plots the empirical probability that nodes at distance *d* become infected on the encounter network (distance on the friendship network: grey; distance on the static encounter network: white).

As the spreading of an infection process depends on the infection rate *β*, we write ${\bar{r}}_{d}\left(\beta \right)$ to compare infection processes with different infection rate. Given a node *s* in the encounter network, we recall that *t*_0_({*s*}) is the first time period in which *s* has an edge (that is, the smallest *t* such that *E*_*u*_(*t*)>0). As we consider infections spreading on the encounter network and distance on the friendship network, we consider seeds that are present in both networks. In each simulation, a single seed is selected uniformly at random between all nodes *s* ∈ |*U*_*F*_∩*U*_*E*_| such that *t*_0_({*s*}) ≤ 500 (as infections on time-varying networks spread for a limited number of time steps, we require them to start early enough). For each *β* ∈ {0, 0.1, 0.25, 0.5} we run 10, 000 simulations. The empirical estimates of ${\bar{r}}_{d}\left(\beta \right)$ for 1 ≤ *d* ≤ 8 are shown in Fig 4 and Table 1.

**Table 1:** Epidemic risk with respect to distance on the friendship network.

*β*	${\bar{r}}_{1}\left(\beta \right)$	${\bar{r}}_{2}\left(\beta \right)$	${\bar{r}}_{3}\left(\beta \right)$	${\bar{r}}_{4}\left(\beta \right)$	${\bar{r}}_{5}\left(\beta \right)$	${\bar{r}}_{6}\left(\beta \right)$	${\bar{r}}_{7}\left(\beta \right)$	${\bar{r}}_{8}\left(\beta \right)$
0.10	4 ⋅ 10^−3^	7 ⋅ 10^−4^	2 ⋅ 10^−4^	7 ⋅ 10^−5^	3 ⋅ 10^−5^	2 ⋅ 10^−5^	1 ⋅ 10^−5^	2 ⋅ 10^−5^
0.25	0.041	0.027	0.014	0.006	0.003	0.003	0.002	0.003 ⋅ 10^−4^
0.50	0.159	0.143	0.095	0.055	0.036	0.031	0.030	0.032
1.00	0.343	0.333	0.262	0.182	0.133	0.118	0.116	0.123

### Predictive accuracy of the friendship network

In order to evaluate how accurately the friendship network predicts epidemic risk at a microscopic level, we consider infection processes initiated at the same seed and spreading independently of each other, and compare the sets of infected nodes. The unpredictability of epidemic risk is due to the structural differences of the different networks as well as to the randomness of the infection processes. Therefore, for each of 5, 000 (a node can be selected multiple times as the seed), we consider four infection processes: two infection processes on the encounter network that spread independently of each other, one on the friendship network, and one on the static version of the encounter network (indexed by *E*_1_, *E*_2_, *F* and *S*, respectively).

For target size *m* and infection *A* ∈ {*E*_1_, *E*_2_, *F*, *S*} initiated at seed *s*, let *t*_*A*_(*m*;*s*) be the first time at which at least *m* nodes are infected (the quantity might be undefined if the infection does not reach at least *m* individuals). When *t*_*A*_(*m*; *s*) is defined, let *I*_*A*_(*m*; *s*) be the corresponding infected set (of size at least *m*). Consider two infections *A*, *B* ∈ {*E*_1_, *E*_2_, *F*, *S*}, *A* ≠ *B*, initiated at the same seed *s* and either spreading on two different networks, or spreading on the same network but independently of each other. Given a target *m*, if both *I*_*A*_(*m*; *s*) and *I*_*B*_(*m*; *s*) are defined, their Jaccard similarity is given by

$$\begin{array}{c} J_{A,B}\left(m;s\right)=\frac{|I_{A}\left(m;s\right)\cap I_{B}\left(m;s\right)|}{|I_{A}\left(m;s\right)\cup I_{B}\left(m;s\right)|}, \end{array}$$

where, given a set *X*, |*X*| denotes its cardinality. These measures allow to characterize how accurately the friendship network and the static version of the encounter network predict epidemic risk on the encounter network (in the SI, we also consider the precision metrics |*I*_*A*_(*m*; *s*) ∩ *I*_*B*_(*m*; *s*)|/|*I*_*A*_(*m*; *s*)| and |*I*_*A*_(*m*; *s*) ∩ *I*_*B*_(*m*; *s*)|/|*I*_*B*_(*m*; *s*)|, which provide similar observations and results).

**Fig 5:**
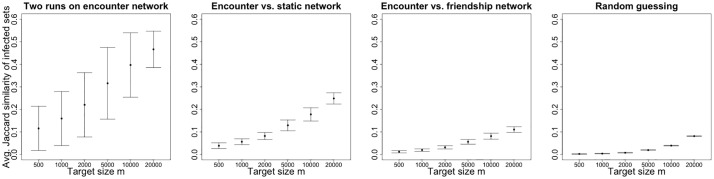
Predictability of a node’s epidemic risk. For each of 5000 selections of a random single seed, two simulations on the encounter network, one on the static network and one on the friendship network are run independently. The average similarities *J*_⋅,⋅_(*m*; *s*) of the infected sets over all seed selections are shown for different pairs of simulations and different target infection size *m* (*x*-axis). First panel: $J_{E_{1},E_{2}}\left(m;s\right)$ (two independent infections on the encounter network). Second panel: *J*_*E*_1_, *S*_(*m*; *s*) (one infection on the encounter network, one infection on the static encounter network). Third panel: *J*_*E*_1_, *F*_(*m*; *s*) (one infection on the encounter network, one infection on the friendship network). Fourth panel: Jaccard similarity between infections on the encounter network and random infected sets of size *m* (nodes belonging to the encounter network). Black points represent the averages of the metrics over all observations such that the metrics are defined, and bars represent standard deviations. Higher values of the *y*-axis correspond to higher prediction accuracy.

Results are shown in Fig 5 and Table 2. Starting from the left, the first panel plots the metrics $J_{E_{1},E_{2}}\left(m;s\right)$ for all seed selections (and a range of values of the target infection size *m*), and represents the baseline unpredictability due solely to the randomness of processes initiated at the same seed and spreading independently on the encounter network. The second panel shows the metrics *J*_*E*_1_, *S*_(*m*; *s*), which includes the unpredictability due to the loss of temporal information in the static version of the encounter network. The third panel shows the metrics *J*_*E*_1_, *F*_(*m*; *s*), which represents the unpredictability of using the friendship network to predict risk on the encounter network. The fourth and rightmost panel shows the Jaccard similarity between infection *E*_1_ and random sets of *m* nodes belonging to but not necessarily connected on encounter network. Such metric represents the admittedly weak baseline of what is achievable by random guessing, without the knowledge of the structure of either the friendship or the encounter network, assuming that only the set of nodes is known. Higher values of the *y*-axis correspond to higher prediction accuracy. For each value of the target *m* separately, $J_{E_{1},E_{2}}\left(m;s\right)$ has larger average than both *J*_*E*_1_, *S*_(*m*; *s*) and *J*_*E*_1_, *F*_(*m*; *s*), and that *J*_*E*_1_, *S*_(*m*; *s*) has larger average than *J*_*E*_1_, *F*_(*m*; *s*). Notably, the intersections of the infected sets on the friendship and encounter networks are substantially and significantly larger than the intersection of random sets (average Jaccard similarity 1.2 ⋅ 10^−2^ vs. 8.3 ⋅ 10^−4^, two-sample t-tests, p-value<2.2 ⋅ 10^−16^). This shows the value of using the friendship network for predicting epidemics risk even if the infection is driven by physical encounter.

**Table 2:** Single seed infection on the encounter network and the static (encounter and friendship) networks. Stochastic infection—Similarity measures.

*m*	$\langle J_{E_{1},E_{2}}\rangle$	〈*J*_*E*_1_, *S*_〉	〈*J*_*E*_1_, *F*_〉	$\langle {\bar{J}}_{E_{1},E_{2}}\rangle$	$\langle {\bar{J}}_{E_{1},S}\rangle$	$\langle {\bar{J}}_{E_{1},F}\rangle$
500	0.115	0.039	0.012	0.270	0.315	0.323
1000	0.159	0.056	0.019	0.325	0.561	0.454
2000	0.220	0.082	0.031	0.438	0.716	0.615
5000	0.316	0.129	0.056	0.571	0.776	0.744
10000	0.397	0.178	0.081	0.664	0.790	0.806
20000	0.466	0.249	0.110	0.788	0.835	0.830

Together, the similarity measures *J*_⋅,⋅_(*m*; *s*) allow to characterize how the randomness of the infection process, the temporal ordering of the encounters and the structural differences between the networks affect the predictability of epidemic risk. Our analyses show that friendship helps identifying the individuals at risk of infection even if the epidemic is driven by physical encounters (compare the third and fourth panels of Fig 5). This is an important result, as in practice it might be feasible to track friendship or other forms of static relationship, but infeasible to track or predict physical encounters. However, knowledge of the friendship network does not allow us to reach the same accuracy as knowing the encounter network (which is usually unavailable or extremely costly to get). On the one hand, the randomness of the infection determines unpredictability of the set of infected individuals, even between independent processes spreading on the encounter network and initiated at the same seed (first panel of Fig 5). On the other hand, structural differences amplify such unpredictability when comparing processes spreading on the friendship network and the encounter network (first and third panels of Fig 5). In addition, our results are not only driven by the static nature of the friendship network opposed to the time-varying nature of the encounter network, as the static version of the encounter network provides more accurate prediction of risk than the friendship network (second and third panels of Fig 5).

### Periodical monitoring and prediction

In addition to the predictive power that knowledge of the friendship network brings on its own, here we show how periodical, even if relatively infrequent, monitoring of the infected population can boost the prediction capabilities of the friendship network. In particular, we show that periodical monitoring of the benchmark infection spreading on the encounter network allows to correct the predicted infection spreading on the friendship network, substantially increasing accuracy. This corresponds to a scenario in which the investigator has knowledge of the friendship network but, in addition, is able to observe the infected population at fixed intervals. Periodical reports of the infection are usually available in the case of real epidemics (e.g., weekly or monthly). After each observation, the set of infected individuals according to the dynamics on the friendship network is updated to match the set of infected individuals according to the dynamics on the encounter network.

Given a seed *s* connected on both the encounter and the friendship networks (and such that *t*_0_(*s*)≤900), we consider an infection spreading on the encounter network and one spreading on the friendship network with periodic corrections (denoted by *F* and *E* respectively), for 500 time steps each and independently of each other. Given an observation window *W*, every *W* time steps the predicted infected set *I*_*F*_(*kW*) on the friendship network is corrected to match the benchmark infected set *I*_*E*_(*kW*) on the encounter network. That is,

$$\begin{array}{c} I_{F}\left(kW\right)=I_{E}\left(kW\right),\;\text{for}\;\text{each}\;k>0. \end{array}$$

and between time *kW* and (*k* + 1)*W* − 1 the set predicted infected *I*_*F*_(*t*) grows according to the ties of the friendship network (because the encounter network, driving the real infection, is not known). We are interested in comparing the sets $I_{E}\left(t\right)$ and *I*_*F*_(*t*) at times *t* = *kW* − 1, that is, right before each correction. Let

$$\begin{array}{c} J_{E,F}\left(k;s,W\right)=\frac{I_{E}\left(kW-1\right)\cap I_{F}\left(kW-1\right)}{I_{E}\left(kW-1\right)\cup I_{F}\left(kW-1\right)}, \end{array}$$

be the Jaccard similarity of the infected sets on the two networks right before a correction (the notation shows its dependence on *W* and on the realization of the infection process, represented by its seed *s*).

**Fig 6:**
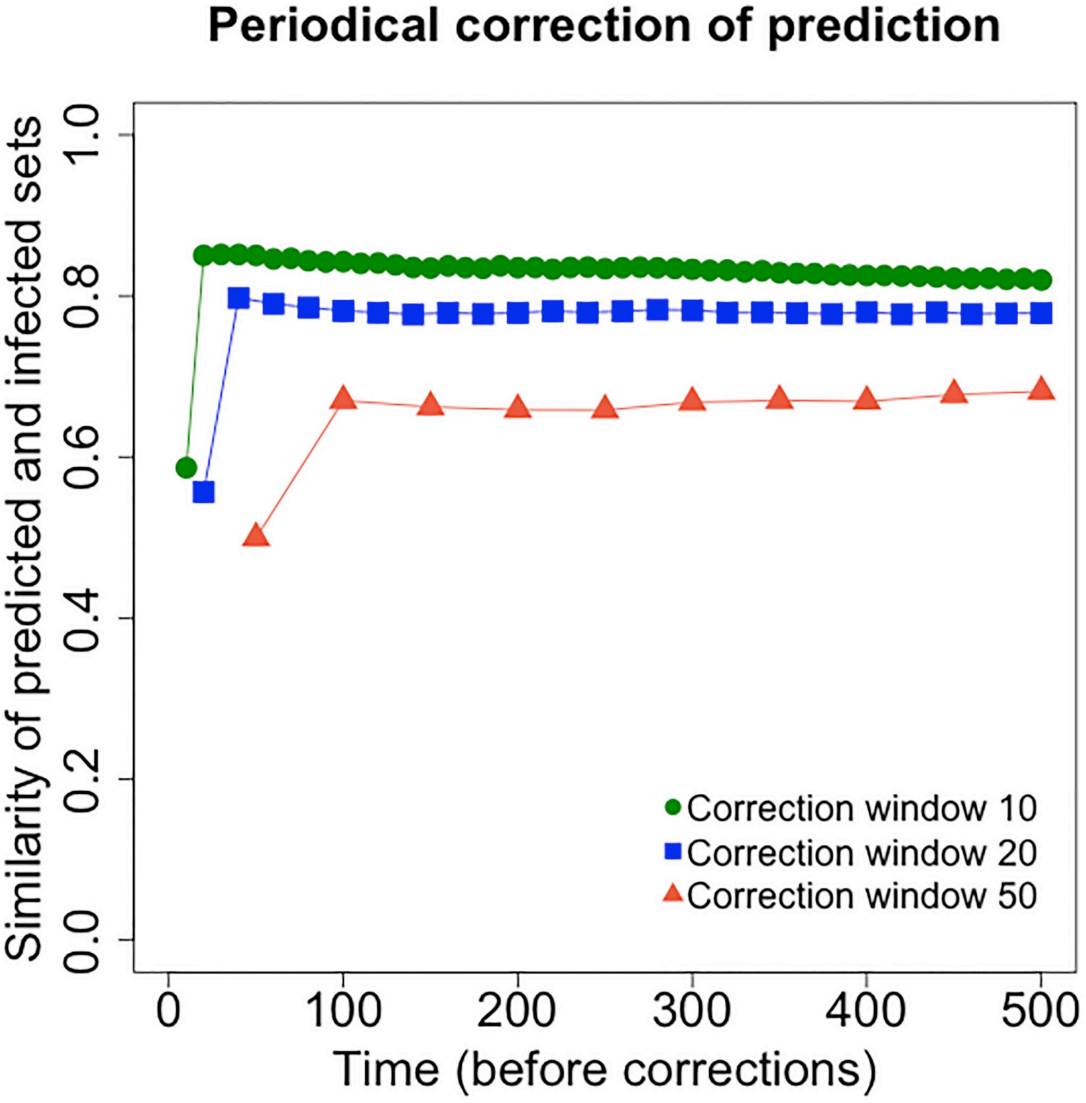
Periodical correction of risk prediction using the friendship network. Shown here is the average Jaccard similarity between the predicted infected sets (according to infections spreading on the friendship network, and periodic corrections) and the infected sets given by infections spreading on the encounter network, before each correction and for different values of the observation window *W*. For each *W* ∈ {10, 20, 50}, 6000 single seeds are selected at random, and for each seed one simulation on the encounter network and one (with periodic corrections) on the friendship network are run. The *x*-axis shows time, the *y*-axis shows Jaccard similarity. Higher values of the *y*-axis correspond to higher prediction accuracy.

Fig 6 plots the average Jaccard similarity of the sets of all infected individuals in the two processes right before each correction (times *kW* − 1, including all previous updates of the predicted infected sets), for window length *W* ∈ {10, 20, 50} (6000 simulations for each *W*). Note that, as each infection process is run for *T* = 500 time steps, the number of corrections (and therefore the number of points in the plots) depends on the choice of *W* and equals *T*/*W*. A high level of prediction accuracy is established early in the process (after the first correction) and maintained over time. The accuracy decreases with larger window size, but even *W* = 50 guarantees good accuracy. Our results suggest that the ability to periodically monitor who is infected (according to the infection on the encounter network) is key to overcome the limits of the friendship network in predicting epidemic risk.

In order to compare all window sizes *W* ∈ {10, 20, 50}, we consider all time steps corresponding to a correction for all choices of *W* and ignore the first correction (i.e., we consider times 100*k* for 1 ≤ *k* ≤ 5). The trend of the average of the Jaccard similarity *J*_*E*,*F*_(*k*; *s*, *W*) with respect to time *t* and window size *W* is captured by a linear relationship. The measure is lower in the case of *W* = 50 (−0.188 with respect to *W* = 10, p-value = 2.74 ⋅ 10^−10^), value for which it increases over time (3.29 ⋅ 10^−3^ every 100 time steps, p-value = 1.27 ⋅ 10^−3^).

### Targeted immunization

In addition to analyzing the power of the online friendship network for real time monitoring during the response phase of an epidemic, we consider as well how it can improve preparedness through immunization campaigns, which can take the form of physical vaccination or information campaigns informing and advocating for safe practices. In this sense, the friendship paradox (i.e., the average friend of an individual is more connected than the average individual [41]) has shown that name-a-friend methods improve the prediction of the peak of an epidemic outbreak [42] and the spread of information online [43]. Instead of considering a scenario in which the same network defines both social ties and infection, here we show that such policies can be effective when social ties are defined according to an online friendship network and infection spreads on an encounter network.

**Fig 7:**
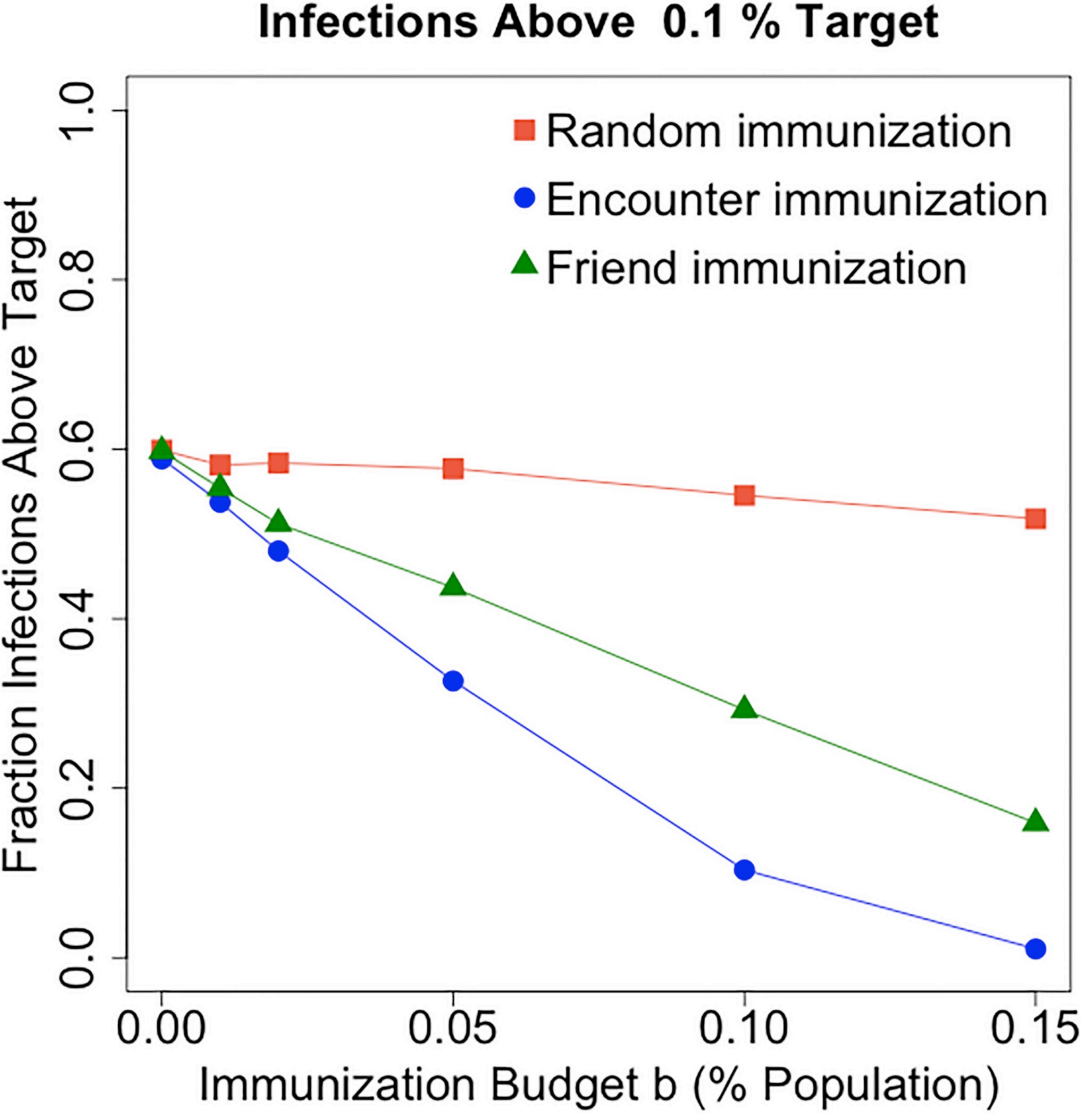
Fraction of infections that do not die out in their early stages as a function of the immunization budget *b* and the immunization method. For each immunization type and *b* ∈ {1%, 2%, 5%, 10%, 15%}, 5000 simulations on the encounter network initiated at random single seeds are run. The *x*-axis shows *b* (% of population that receives immunization), the *y*-axis shows the fraction of infections whose final size is above 0.1% of the entire population (taken as an indicator that an infection did not die out in its early stages). Lower values of the *y*-axis correspond to more effective immunization strategies.

**Fig 8:**
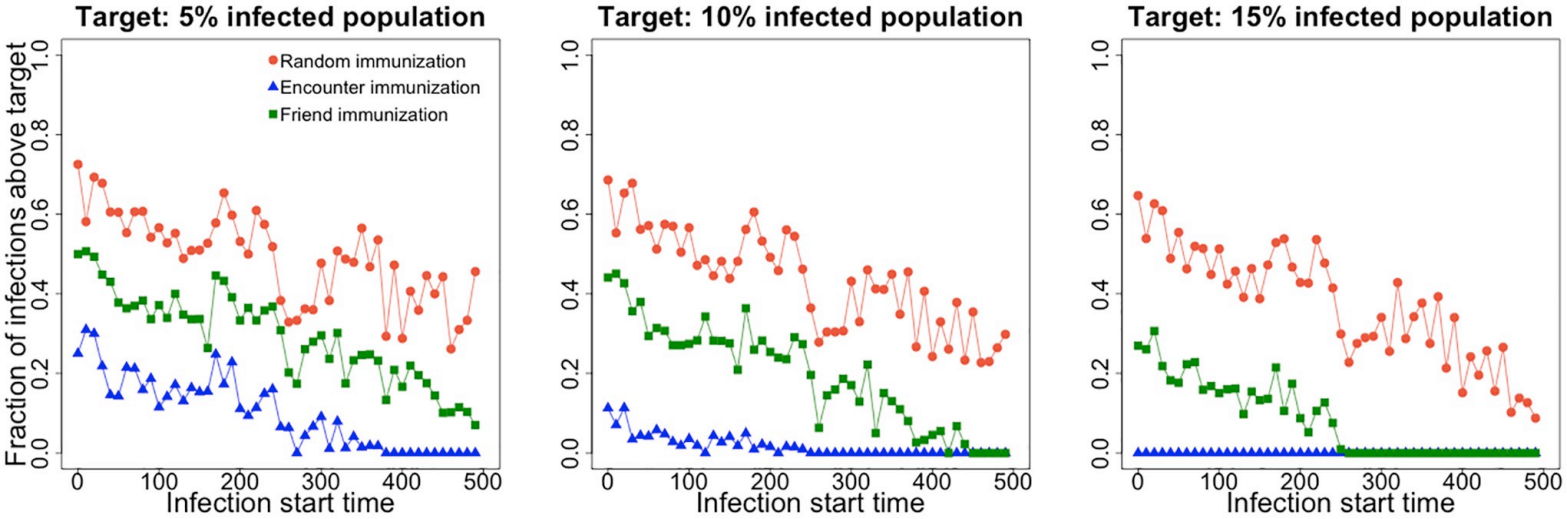
Final infection size as a function of the immunization method and the infection start time. Given immunization budget *b* = 5% of the entire population, for each immunization type, 5000 simulations on the encounter network are initiated at random single seeds. Each panel considers a target infection size expressed as a percentage of the entire population. The *x*-axis shows the infection start time *t*_0_(*s*) of seed *s*, the *y*-axis shows the fraction of infections whose final size is above the given target. Lower values of the *y*-axis correspond to more effective immunization strategies.

We consider a scenario in which a fixed budget is available for immunization (e.g., limited amount of vaccine) and must be effectively allocated in order to contain an epidemic spreading on the encounter network. In contrast to purely random immunization (where target individuals to immunize are selected at random), we consider a strategy that selects random friends of randomly chosen individuals for immunization (friend immunization). The *friend immunization* method selects target individuals to immunize as follows: (i) select a set *R* of *n* random individuals; (ii) for each individual *x* ∈ *R*, randomly select a friend, that is, an individual *y* such that *x* and *y* are connected in the friendship network; (iii) each individual *y* receives immunization. The friend immunization method results in a more effective use of the immunization budget, substantially increasing the probability that an infection dies out in its early stages (Fig 7) and strongly reducing the final infection size (Fig 8) with respect to random immunization. Moreover, it only requires a small additional cost (in terms of the number of immunized individuals) to obtain the same effect as an ideal strategy that targets future encounters rather than friends (encounter immunization). The *encounter immunization* selection method is similarly to the friend immunization method, with the difference that for each *x* ∈ *R* a future encounter *y* is selected.

Immunization budget is expressed as a fraction *b* of the entire population. Once the network size is fixed, considering immunization budget in terms of a fraction *b* of the entire population is equivalent to setting a fixed number of individual to target (e.g. a fixed number of vaccine). We represent *b* as a fraction for representation purposes, in order to stress that an immunization budget that is small relative to the population can be effectively allocated. For *b* ∈ {1%, 2%, 5%, 10%, 15%}, Fig 7 shows the fraction of infections above 0.1% of the entire population as a function of *b* for all considered immunization methods (5000 simulations for each immunization method and value of *b*). We consider a 0.1% target for the final infection as an indicator that the infection did not die out in its early stages. Lower values of the *y*-axis correspond to more effective immunization strategies. The trend in Fig 7 is captured by a linear model with interactions between immunization type and immunization budget (*R*^2^ = 0.98). Each 1% increase of the immunization budget determines a 0.5% decrease of the fraction of infections above the 0.1% target for random immunization (p-value = 0.0299), an additional 2.36% decrease for friend immunization (p-value = 2.77 ⋅ 10^6^), and an additional 3.5% decrease for encounter immunization (p-value = 4.03 ⋅ 10^8^). Despite its simplicity, friend immunization provides comparable effectiveness as the encounter immunization strategy (which would require knowledge of the encounter network), at a small additional cost. For a fixed value of the *y*-axis, observe the limited extra immunization budget required to reach that performance employing friend immunization rather than encounter immunization.

Regarding the size of the infected population, Fig 8 shows (for *b* = 5%) the fraction of infections that reach given target sizes (5%, 10%, 15% of the entire population in the left, middle and right panel respectively) as a function of the infection start time *t*_0_(*s*) of the seed for all immunization methods (5000 simulations for each immunization method). The *y*-axis shows the fraction of infections whose final size is above the given target, and lower values correspond to more effective immunization strategies. Friend immunization provides a large advantage with respect to random immunization, and its effectiveness increases with increasing immunization budget faster than it does for the latter.

## Discussion

Epidemics are complex problems that draw tremendous efforts from Governments and International Organizations. Given the diversity of contexts in which they happen and the varied nature of different diseases, epidemic response presents multiple challenges that need to be addressed in order to curve the thread. In addition, an increasingly connected world has shown in the last decades the fast pace at which epidemics can turn into pandemics—see for example the H1N1 crisis of 2009, the Ebola outbreak in 2014, or the Zika epidemic of 2015. The increased speed and reach have further pointed out the need to develop more and better tools to target resources in a more accurately, timely and efficient manner.

One key aspect of epidemic response is forecasting of risk, that is, predicting what individuals or areas are at higher risk of being infected in the future. Within the United Nations this need translated into diverse efforts, ranging from contact tracing methodologies [45] that are critical in highly contagious outbreaks such as Ebola, to the implementation of tools that combine classical statistical models with meteorological, entomological and epidemiological data (used for example in the early detection of Dengue outbreaks [46]), to the promotion of usage of new sources of data [47].

These approaches are not intended to substitute one another, but they reflect the complexity of predicting and containing epidemic risk and the need to target the particular idiosyncrasies of the situation at hand. Contact tracing, for example is one of the most effective methods for containing an epidemic, but requires huge effort and cost to identify cases, perform interviews and monitor contacts. In addition, it relies on active participation and cooperation by the affected communities [45]. Phone call data on the other hand is a less expensive source of real time information and has proven useful during epidemic outbreaks such as the West African Ebola outbreak [48], but accessing call records presents important privacy issues, even when anonymized, and aggregation poses certain limitations when predicting the geographical spread of an epidemic [49, 50].

On-line friendship networks are publicly available, partially or fully, on many online platforms. In a practical scenario, the ego-centric friendship network of an infected individual could be quickly accessed with the individual’s cooperation. Therefore, online social networks presents themselves as an additional data resource to inform epidemic response in contexts where contact information is hard to retrieve. This paper is a first step in this direction, exploring how the knowledge of a friendship or other social network, while not being the physical avenue of epidemic spread, can be operationalized to help predict and contain epidemic risk.

It is perhaps not surprising that the friendship network provides useful information for identifying the individuals at risk even if the infection spreads on the encounter network. However, due to the structural differences between the two networks, accuracy of predictions using the friendship network does not come close to the ideal case in which the encounter network is known. Despite these differences in the networks and the prediction results, our simulations show that knowledge of the friendship network enables effective monitoring and immunization strategies. Very high prediction accuracy using the friendship network can be reached and maintained if periodical yet infrequent reports of the infected population are available, as they are in many real epidemic response scenarios. In addition, in the context of immunization with limited budget, simply asking individuals to name a friend enables the effective use of the available resources, and requires a small additional investment to reach the same performance as knowing the encounter network.

When it is known who is infected or likely to become infected (e.g., individuals traveling to certain countries who might have come in contact with a pathogen), accurate prediction of the individuals at risk of contagion would allow targeted monitoring and immunization. Taken together, our results highlight the opportunity of using a friendship network for predicting, monitoring and containing epidemics. In real scenarios, friendship, family or professional networks (which can be considered static or almost static) are more likely to be available than time-varying networks of physical encounters, which would require extensive tracking of the population. In addition, the encounter network is fully accessible only in a context of “prediction in retrospect”, as in the case of the present work. Information to predict future encounters between individuals is likely to be unavailable, at least at a detailed level. However, a feasible approach could use past encounters as a proxy of future encounters. In fact, it is known that human mobility and encounter present high spatial and temporal regularity and predictability [51–54]. From a practical perspective, networks based on social relationships (such as a friendship network) might be complemented by information about past encounter. In particular, the links in a friendship network (which might be initialized according to known or self-reported familial or professional ties) could be updated based on past encounters in order to reflect the encounter network in an increasingly accurate fashion. Such approaches could be complimentary to the periodical monitoring of the infected population that we considered in the paper, and represent an interesting avenue of future research.

In the present paper we assumed that the network structure does not change over time based on an infection. For example, certain infected individuals might avoid contact with others, and thus be removed from the encounter network, preventing additional infection. Removal might happen with a given probability or with some delay from the time of infection. We leave the investigation of such scenarios to future research, and focused on the simpler and fundamental scenario in which infected individuals remain in the network.

We considered reviews as a proxy of physical encounter—an edge is active between two users on day *t* if they both posted a review to or a tip about the same business on day *t*. This constitutes an approximation of real physical encounter, which would require users to visit (rather than review) a business at about the same time. In order to justify this assumption, we observe that the time of a review is a proxy of the time of the visit to a business, and that infections do not necessarily require direct physical contact. In fact, in the case of certain airborne diseases, particles can remain suspended in the air for several hours after an infected individual has been in a room [44]. In the context of our dataset, after an infected user visits a business, the infection might spread to customers who visit the business later in the day. Other proxies of physical encounter, such as proximity measured by Bluetooth devices, are usually limited to small population, and suffer different limitations (e.g., the signal passes through walls).

Our simulations are based on a large dataset that allowed us to build a static friendship network and a time-varying encounter network that is a candidate vehicle for the spread of a pathogen. The dataset includes more than 100k individuals and spans more than 4 years of activity. In general, other datasets might be available and allow similar analyses. Friendship networks whose edges have a different semantic than that considered in the present work might lead to different observations.

Epidemic response is a complex and often time critical problem, requiring from the research community to help better understand what sources of data and methodologies can help shed light and better target efforts in real world scenarios. This work shows how friendship networks can be used as a valuable resource when coupled with periodically available case data. To further this line of research it is important to count with more and more comprehensive datasets that include information on contact/mobility as well as on friendship/relation, ideally during the course of an epidemic. It is also important, when designing innovative methodologies for containing and predicting epidemic risk, to closely consider the processes and data followed and gathered, respectively, by governments and humanitarian organizations on the ground when responding to epidemics as an important asset to improve the accuracy of and find value in alternative methods and data.
